# Supplementary material for: Heat adaptation of phage T7 under an extended genetic code
Source: Virus Evol. 2021 Dec 1;7(2):veab100. doi: 10.1093/ve/veab100 (PMC8923235; doi:10.1093/ve/veab100)
Supplement: veab100_Supp [file veab100_supp.zip › Sequencing_Analysis.zip!/Sequencing_Analysis/S1/evidence/JC_SIDE_2_55.html]

BRESEQ :: Evidence


| New junction evidence | | | | | | | | | | | |
| --- | --- | --- | --- | --- | --- | --- | --- | --- | --- | --- | --- |
|  | | seq id | position | reads (cov) | reads (cov) | score | skew | freq | annotation | gene | product |
| \* | ? | NC\_001604.1 | = 13578 | 10 (0.000) | 121 (0.030) | 37/584 | 31.3 | 93.5% | intergenic (–/–) | *–/–* | –/– |
|  | ? | NC\_001604.1 | 14348 = | 7 (0.000) |  |  |  |  | intergenic (–/–) | *–/–* | –/– |
| Rejected: Coverage evenness skew score above cutoff. | | | | | | | | | | | |

Only 100 of 4642 total aligned reads displayed.  

`GGACAAGACAGACTGGCTGGAAGAAACCAGAAGTTACCAAGCTGAGTCATAAGGTGTTCGATAAGGACACTATGACCCACATCAAGGCTGGTGATTGGGTTAAGGTTGACATGGGAGTTGTTGGTGGATACGGCTACGTCCGCTCAGTTAGTGGCAAATATGCACAAGTGTCATACATCACAGGTGTTACTCCACGCGGTGCAATCGTTGCCGATAAGACCAACATGATTCACACAGGTTTCTTGACAGTTGTTTCATATGAAGAGATTGTTAAGTCACGATAATCAATAGGAGAAATCAATATGATCGTTTCTGACATCGAAGCTAACGCCCTCTT‑‑AGAGAGCGTCACTAAGTTCCACTGCGGGGTTATCTACGACTACTCCACCGCTGAGTACGTAAGCTACCGTCCGAGTGACTTCGGTGCGTATCTGGATGCGCTGGAAGCCGAGGTTGCACGAGGCGGTCTTATTGTGTTCCACAACGGTCACAAGTATGACGTTCCTGCATTGACCAAACTGGCAAAGTTGCAATTGAACCGAGAGTTCCACCTTCCTCGTGAGAACTGTATTGACACCCTTGTGTTGTCACGTTTGATTC  >  NC_001604.1/14051‑14647`  

                                                                                                                                                                                                                                                                                                         `|`                                                                                                                                                                                                                                                                                                               
`ctttccagacgactactagaagtttcgctggagagtcccattctaatacgactcactaaaggagacacaccatgttcaaactgattaagaagttaggccaactgctggttcgtatgtacaacgtggaagccaagcgactgaacgatgaggctagtaaagaggcaacacattcacgcgctctggcgattcgctccaacgaactggctgacagtgcatccactatagttaccgaggctgcccgtgtggcaaaccaagctacacagctttccaaatcctttgagtaatccaTAGGAGAAATCaa                                                                                                                                                                                                                                                                                                            >  1:35944‑M2/289‑301 (MQ=255)`  
`ctttccagacgactacgagaagtttcgctggagagtcccattctaatacgactcactaaaggagacacaccatgttcaaactgattaagaagttaggccaactgctggttcgtatgtacaacgtggaagccaagcgactgaacgatgaggctcgtaaagaggccacacagtcacgcgctctggcgattcgctccaacgaactggctgacagtgcatccactaaagttaccgaggctgcccgtgtggcaaaccaagctcaacagctttccaaattctttgagtaatcaaTAGGAGAAatcct                                                                                                                                                                                                                                                                                                            >  1:576330‑M2/289‑299 (MQ=255)`  
   `tccagacgactacgagaagtttcgctggagagtcccattctaatacgactcactaaaggagacacaccatgttcaaactgattaagaagttaggccaactgctggttcgtatgtacaacgtggaagccaagcgactgaacgatgaggctcgtaaagaggccacacagtcacgcgctctggcgattcgctccaacgaactggctgacagtgcatccactaaagttaccgaggctgcccgtgtggcaaaccaagctcaacagctttccaaattctttgagtaatcaaTAGGAGAAATCAatat                                                                                                                                                                                                                                                                                                         <  1:608741‑M2/16‑1 (MQ=255)`  
   `tccagacgactacgagaagtttcgctggagagtcccattctaatacgactcactaaaggagacacaccatgttcaaactgattaagaagttaggccaactgctggttcgtatgtacaacgtggaagccaagcgactgaacgatgaggctcgtaaagaggccacacagtcacgcgctctggcgattcgctccaacgaactggctgacagtgcatccactaaagttaccgaggctgcccgtgtggcaaaccaagctcaacagctttccaaattctttgagtaatcaaTAGGAGAAATCAatat                                                                                                                                                                                                                                                                                                         <  1:347132‑M2/16‑1 (MQ=255)`  
           `ctacgagaagtttcgctggagagtcccattctaatacgactcactaaaggagacacaccatgttcaaactgattaagaagttaggccaactgctggttcgtatgtacaacgtggaagccaagcgactgaacgatgaggctcgtaaagaggccacacagtcacgcgctctggcgattcgctccaacgaactggctgacagtgcatccactaaagttaccgaggctgcccgtgtggcaaaccaagctcaacagctttccaaattctttgagtaatcaaaCAGGAGAAATCAATATGATCGttt                                                                                                                                                                                                                                                                                                 >  1:100160‑M2/278‑301 (MQ=255)`  
                        `tcgctggagagtcccattctaatacgactcactaaaggagacacaccatgttcaaactgattaagaagttaggccaactgctggttcgtatgtacaacgtggaagccaagcgactgaacgatgaggctcgtaaagaggccacacagtcacgcgctctggcgattcgctccaacgaactggctgacagtgcatccactaaagttaccgaggctgcccgtgtggcaaaccaagctcaacagctttccaaattctttgagtaatcaaTAGGAGAAATCAATATGATCGTTTCTgacatctttct                                                                                                                                                                                                                                                                                    >  1:68560‑M2/265‑296 (MQ=255)`  
                        `cgctggagagtcccattctaatacgactcactaaaggagacacaccatgttcaaactgattaagaagttaggccaactgctggttcgtatgtacaacgtggaagccaagcgactgaacgatgaggctcgtaaagaggccacacagtcacgcgctctggcgattcgctccaacgaactggctgacagtgcatccactaaagttaccgaggctgcccgtgtggcaaaccaagctcaacagctttccaaattctttgagtaatcaaaCAGGAGAAATCAATATGATCGTTTCTGACATCGAAGc                                                                                                                                                                                                                                                                                    >  1:65375‑M2/265‑301 (MQ=255)`  
                                 `gtcccattctaatacgactcactaaaggagacacaccatgttcaaactgattaagaagttaggccaactgctggttcgtatgtacaacgtggaagccaagcgactgaacgatgaggctcgtaaagaggccacacagtcacgcgctctggcgattcgctccaacgaactggctgacagtgcatccactaaagttaccgaggctgcccgtgtggcaaaccaagctcaacagctttccaaattctttgagtaatcaaaCAGGAGAAATCAATATGATCGTTTCTGACATCGAAGCTAACgcccc                                                                                                                                                                                                                                                                           >  1:214075‑M2/256‑300 (MQ=255)`  
                                 `gtcccattctaatacgactcactaaaggagacacaccatgttcaaactgattaagaagttaggccaactgctggttcgtatgtacaacgtggaagccaagcgactgaacgatgaggctcgtaaagaggccacacagtcacgcgctctggcgattcgctccaacgaactggctgacagtgcatccactaaagttaccgaggctgcccgtgtggcaaaccaagctcaacagctttccaaattctttgagtaatcaaaCAGGAGAAATCAATATGATCGTTTCTGACATCGAAGCTAACGCCct                                                                                                                                                                                                                                                                           >  1:583297‑M2/256‑301 (MQ=255)`  
                                  `gtcccattataaaacgactcaataaaggagacacaccacgttcaaactgaataagaaggtaggccaactgctggttcgtatgtacaacgtggaagccaagcgactgaacgatgaggctcgtaaagaggccacacagtcacgcgctctggcgattcgctccaacgaactggctgacagtgcatccactaaagttaccgaggctgcccgtgtggcaaaccaagctcaacagctttccaaatgctttgagtaatcaaTAGGAGAAATCAATATGATCGTTTCTGACATCGAAGCTAACGCCctc                                                                                                                                                                                                                                                                          <  1:542127‑M2/47‑1 (MQ=255)`  
                                      `attctaatacgactcactaaaggagacacaccatgttcaaactgattaagctgttaggccaccttctggttcgtatgtacaacttggaagtcaagcgcttaaacgatgagcctcgtaaagtggcctcacagtcacgcgttctggcgattcgctacaacgaactagctgctagtgcatccactaaagttaccgaggatgcccgtgtggcaaaccaagctcaacagctttccaaattctttgagtaatcaaaCTGGAGAAATCAATATGATCGTTTCTGACATCGAAGCTAAAGCCCTCTT‑‑ag                                                                                                                                                                                                                                                                    >  1:219696‑M2/251‑301 (MQ=255)`  
                                       `ttctaatacgactcactaaaggagacacaccatgttcaaactgattaagaagttaggccaactgctggttcgtatgtacaacgtggaagccaagcgactgaacgatgaggctcgtaaagaggccacacagtcacgcgctctggcgattcgctccaacgaactggctgacagtgcatccactaaagttaccgaggctgcccgtgtggcaaaccaagctcaacagctttccaaattctttgagtaatcaaaCAGGAGAAATCAATATGATCGTTTCTGACATCGAAGCTAACGCCCTCTT‑‑aga                                                                                                                                                                                                                                                                   >  1:225152‑M2/250‑301 (MQ=255)`  
                                        `ttctaatacgactcactaaaggagacacaccatgttcaaactgattaagaagttaggccaactgctggttcgtctgtacaacatgtaagccaagcgactgaacgatgaggctcgtaaagaggccacacagtcacgcgatctggcgcttcgctccaccgaactggctgacagttcatccactaactttacctaggctgcccgtgtggcaaaccaagctcaacagctttccaaattctttgagtagtcaaTAGGAGAACTCAATATTATCGTTTCTGACATCGAAGCTAACGCCCTCTT‑‑agag                                                                                                                                                                                                                                                                  >  1:561590‑M2/249‑301 (MQ=255)`  
                                        `ttctaatacgactcactaaaggagacacaccatgttcaaactgattaagaagttaggccaactgctggttcgtatgtacaacgtggaagccaagcgactgaacgatgaggctcgtaaagaggccacacagtcacgcgctctggcgattcgctccaacgaactggctgacagtgcatccactaaagttaccgaggctgcccgtgtggcaaaccaagctcaacagctttccaaattctttgagtaatcaaTTGGAGAAATCAATATGATCGTTTCTGACATCGAAGCTAACGCCCTCTT‑‑agag                                                                                                                                                                                                                                                                  >  1:196424‑M2/249‑301 (MQ=255)`  
                                        `ttctaatacgactcactaaaggagacacaccatgttcaaactgattaagaagttaggccaactgctggttcgtatgtacaacgtggaagccaagcgactgaacgatgaggctcgtaaagaggccacacagtcacgcgctctggcgattcgctccaacgaactggctgacagtgcatccactaaagttaccgaggctgcccgtgtggcaaaccaagctcaacagctttccaaattctttgagtaatcaaTAGGAGAAATCAATATGATCGTTTCTGACATCGAAGCTAACGCCCTCTT‑‑agag                                                                                                                                                                                                                                                                  >  1:165482‑M2/249‑301 (MQ=255)`  
                                        `ttctaatacgactcactaaaggagacacaccatgttcaaactgattaagaagttaggccaactgctggttcgtaggtacaacgtggaagccaagcgactgaacgatgaggctcgtaaagaggccacacagtcacgcgctctggcgattcgctccaacgaactggctgacagtgcatccactaaagttaccgaggctgcccgtgtggcaaaccaagcacaacagctttccaaattctttgagtaatcaaTAGGAGAAATCAATATGATCGTTTCTGACATCGAAGCTAACGCCCTCTT‑‑agag                                                                                                                                                                                                                                                                  <  1:149306‑M2/53‑1 (MQ=255)`  
                                        `ttctaatacgactcactaaaggaaacacaccatgttcaaactgattaagaagttaggccaactgctggttcgtatttacaacgtggaagccaagcgactgaacgatgaggctcgtaaagaggccacacagtcacgcgctctggcgattcgctccaacgaactggctgacagtgcatccactaaagttaccgaggctgcccgtgtggcaaaccaagctcaacagctttccaaattctttgagtaatcaaTAGGAGAAATCAATATGATCGTTTCTGACATCGAAGCTAACGCCCTCTT‑‑agag                                                                                                                                                                                                                                                                  <  1:520014‑M2/53‑1 (MQ=255)`  
                                        `ttctaataagactcactaaaggagaaacaccatgtgcaaactgattaagaagttaggccaactgctggttcgtagatacaacgtggaagccaagcgactgaacgatgaggctcgtaaagaggccacacagacacgcgctctggcgattcgctccaacgaactggctgaaagtgcatccactaaagttacagaggatgcccgtgtggcaaaccaagctcaacagctttccaaattctttgagtaatcaaTAGGAGAAATCAATATGATCGTTTCTGACATCGAGGCTAACGCCCTCTT‑‑agag                                                                                                                                                                                                                                                                  <  1:619558‑M2/53‑1 (MQ=255)`  
                                        `tctaatacgtctcactaaaggagacacaccatgttcaaactgattaagaagttaggccaactgctggttcgtatgtacaacgtggaagccaagcgactgaacgatgaggctcgtaaagaggccacacagtcacgcgctctggcgattcgctccaacgaactggctgacagtgcatccactaaagttaccgaggctgcccgtgtggcaaaccaagctcaacagctttccaaattctttgagtaatcaaaCAGGAGAAACCATTATGATCGTTTCTGACATCGAAGCTAACGCCCTCTT‑‑agag                                                                                                                                                                                                                                                                  <  1:143409‑M2/53‑1 (MQ=255)`  
                                        `tctaatacgactcaataaaggagacacaccatgttcaaactgattaagaagttaggccaactgctggttcgtatgtacaacgtggaagccaagcgactgaacgatgaggctcgtaaagaggccacacagtcacgcgctctggcgattcgctccaacgaactggctgacagtgcatccactaaagttaccgaggctgcccgtgtggcaaaccaagctcaacagctttccaaattctttgagtaatcaaaCAGGAGAAATCAATATGATCGTTTCTGACATCGAAGCTAACGCCCTCTT‑‑agag                                                                                                                                                                                                                                                                  <  1:441907‑M2/53‑1 (MQ=255)`  
                                        `tataagataaagcactaaaggagacaaaccatgttcaaactgagtaagaagttaggccaactgctggttcgtatgtaaaacgtggaagccaagcgactgaacgatgaggctcgtaaagaggccacacagtcacgcgctctggcgattcgctccaacgaactggctgacagtgcatccactaaagttaccgaggctgcccgtgtggcaaaccaagctcaacagctttccaaattctttgagtaatcaaaCATGAGAAACCATTATGATCGTTTCTGACATCGAAGCTAACGCCCTCTT‑‑agag                                                                                                                                                                                                                                                                  <  1:375318‑M2/53‑1 (MQ=255)`  
                                        `atgtgtataagagacagaaaggagacacaccatggtcaaactgattaagaagttaggccaactgctggttcgtatgtacaacgtggaagccaagcgactgaacgatgaggctcgtaaagaggccacacagtcacgcgctctggcgattcgctccaacgaactggctgacagtgcatccactaaagttaccgaggctgcccgtgtggcaaaccaagctcaacagctttccaaattctttgagtaatcaaTAGGAGAAATCAATATGATCGTTTCTGACATCGAAGCTAACGACCTCTT‑‑agag                                                                                                                                                                                                                                                                  <  1:260825‑M2/53‑1 (MQ=255)`  
                                            `aatacgactcactaaaggagacacaccatgttcaaactgattaagaagttaggccaactgctggttcgtatgtacaacgtggaagccaagcgactgaacgatgaggctcgtaaagaggccacacagtcacgcgctctggcgattcgctccaacgaactggctgacagtgcatccactaaagttaccgaggctgcccgtgtggcaaaccaagctcaacagctttccaaattctttgagtaatcaaTAGGAGAAATCAATATGATCGTTTCTGACATCGAAGCTAACGCCCtctt‑‑agagctgt                                                                                                                                                                                                                                                              >  1:93433‑M2/245‑297 (MQ=255)`  
                                                    `cactaaaggagacacaccatgttcaaactgattaagaagttaggccaactgctggttcgtatgtacaacgtggaagccaagcgactgaacgatgaggctcgtaaagaggccacacagtcacgcgctctggcgattcgctccaacgaactggctgacagtgcatccactaaagttaccgaggctgcccgtgtggcaaaccaagctcaacagctttccaaattctttgagtaatcaaaCAGGAGAAATCAATATGATCGTTTCTGACATCGAAGCTAACGCCCTCTT‑‑AGAGAGCGTCACTAAg                                                                                                                                                                                                                                                      >  1:208293‑M2/237‑301 (MQ=255)`  
                                                    `cactaaaggagacacaccatgttcaaactgattaagaagttaggccaactgctggttcgtatgtacaacgtggaagccaagcgactgaacgatgaggctcgtaaagaggccacacagtcacgcgctctggcgattcgctccaacgaactggctgacagtgcatccactaaagttaccgaggctgcccgtgtggcaaaccaagctcaacagctttccaaattctttgagtaatcaaaCAGGAGAAATAAATATGAACGTTTCTGACATCGAAGCTAACGCCCTCTT‑‑AGAGAGCGTCACTAAg                                                                                                                                                                                                                                                      >  1:514779‑M2/237‑301 (MQ=255)`  
                                                        `gtgtataagagacagtgttcaaactgattaagaagttaggccaactgctggttcgtatgtacaacgtggaagccaagcgactgaacgatgaggctcgtaaagaggccacacagtcacgcgctctggcgattcgctccaacgaactggctgacagtgcatccactaaagttaccgaggctgcccgtgtggcaaaccaagctcaacagctatccaaattctttgagtaatcaaaCAGGAGAAACCATTATGATCGTTTCTGACATCGAAGCTAACGCCCTCTT‑‑AGAGAGCGTCACTAAGTTcc                                                                                                                                                                                                                                                  <  1:368914‑M2/69‑1 (MQ=255)`  
                                                          `gggagacacaccatgttcaaactgattaagaagttaggccaactgctggttcgtatgtacaacgtggaagccaagcgactgaacgatgaggctcataaagaggccacacagtcacgcgctctggcgattcgctccaacgaactggctgacagtgcatccactaaagttaccgaggatgcccgtgtggcaaaccaagctcaacagctttccaaattctttgagtgatcaaaCAGGAGAAATCAATATGATCGTTTCTGACATCGAAGCTAACGCCCTCTT‑‑AGAGAGCGTCACTAAGTTCCAc                                                                                                                                                                                                                                                <  1:116672‑M2/71‑1 (MQ=255)`  
                                                          `aggagacacaccatgttcaaactgattaagaagttaggccaactgctggttcgtatgtacaacgtggaagccaagcgactgaacgatgaggctcgtaaagaggccacacagtcacgcgctctggcgattcgctccaacgaactggctgacagtgcatccactaaagttaccgaggctgcccgtgtggcaaaccaagctcaacagctttccaaattctttgagtaatcaaaCAGGAGAAATCAATATGATCGTTTCTGACATCGAAGCTAACGCCCTCTT‑‑AGAGAGCGTCACTAAGTTCCAc                                                                                                                                                                                                                                                >  1:64855‑M2/231‑301 (MQ=255)`  
                                                            `gagacacaccatgttcaaactgattaagaagttaggccaactgctggttcgtatgtacaacgtggaagccaagcgactgaacgatgaggctcgtaaagaggccacacagtcacgcgctctggcgattcgctccaacgaactggctgacagtgcatccactaaagttaccgaggctgcccgtgtggcaaaccaagctcaacagctttccaaattctttgagtaatcaaaCAGGAGAAATCAATATGATCGTTTCTGACATCGAAGCTAACGCCCTCTT‑‑AGAGAGCGTCACTAAGTTCCACTg                                                                                                                                                                                                                                              >  1:61486‑M2/229‑301 (MQ=255)`  
                                                                 `aacaccatgttcaaactgattaagaagttaggccaactgctggttcgtatgtacaacgtggaagccaagcgactgaacgatgaggctcgtaaagaggccacacagtcatgcgctctggcgattcgctccaacgaactggctgacagtgcatccactaaagttaccgaggctgcccgtgtggcaaaccaagctcaacagctttccaaattctttgagtaatcaaTAGGAGAAATCAATATGATCGTTTCTGACATCGAAGCTAACGCCCTCTT‑‑AGAGAGCGTCACTAAGTTCCACTGCgggg                                                                                                                                                                                                                                         <  1:273091‑M2/78‑1 (MQ=255)`  
                                                                        `tgttcaaactgattaagaagttaggccaactgctggttcgtatgtacaacgttgaagccaagcgactgaacgatgaggctcgtaaagaggccacacagtcacgcgctctggcgattcgctccaacgaactggctgacagtgcatccactaaagttaccgaggctgcccgtgtggcaaaccaagctcaacagctttccaaattctttgagtaatcaaTAGGAGAAATCAATATGATCGTTTCTGACATCGAAGCTAACGCCCTCTT‑‑AGAGAGCGTCACTAAGTTCCACTGCGGGGTTATCTa                                                                                                                                                                                                                                  <  1:353158‑M2/85‑1 (MQ=255)`  
                                                                         `gttcaaactgattaagaagttaggccaactgctggttcgtatgtacaacgtggaagccaagcgactgaacgatgaggctcgtaaagaggacacacagtcacgcgctctggcgattcgctccaacgaactggctgacagtgcatccactaaagttaccgaggctgcccgtgtggcaaaccaagctcaacagctttccaaattctttgagtaatcaaTAGGAGAAATCAATATGATCGTTTCTGACATCGAAGCTAACGCCCTCTT‑‑AGAGAGCGTCACTAAGTTCCACTGCGGGGTTATCTAc                                                                                                                                                                                                                                 >  1:110686‑M2/216‑301 (MQ=255)`  
                                                                            `caaactgattaagaagttaggccaactgctggttcgtatgtacaacgtggaagccaagcgactgaacgatgaggctcgtaaagaggccacacagtcacgcgctctggcgattcgctccaacgaactggctgacagtgcatccactaaagttaccgaggctgcccgtgtggcaaaccaagctcaacagctttccaaattctttgagtaatcaaTAGGAGAAATCAATATGATCGTTTCTGACATCGAAGCTAACGCCCTCTT‑‑AGAGAGCGTCACTAAGTTCCACTGCGGGGTTATCTACGac                                                                                                                                                                                                                              <  1:154796‑M2/89‑1 (MQ=255)`  
                                                                            `caaactgattaagaagttaggccaactgctggttcgtatgtacaacgtggaagccaagcgactgaacgatgaggctcgtaaagaggccacacagtcacgcgctctggcgattcgctccaacgaactggctgacagtgcatccactaaagttaccgaggctgcccgtgtggcaaaccaagctcaacagctttccaaattctttgagtaatcaaTAGGAGAAATCAATATGATCGTTTCTGACATCGAAGCTAACGCCCTCTT‑‑AGAGAGCGTCACTAAGTTCCACTGCGGGGTTATCTACGac                                                                                                                                                                                                                              <  1:279747‑M2/89‑1 (MQ=255)`  
                                                                                `ctgattaagaagttaggccaactgctggttcgtatgtacaacgtggaagccaagcgactgaacgatgaggctcgtaaagaggccacacagtcacgcgctctggcgattcgctccaacgaactggctgacagtgcatccactaaagttaccgaggctgcccgtgtggcaaaccaagctcaacagctttccaaattctttgagtaatcaaTAGGAGAAATCAATATGATCGTTTCTGACATCGAAGCTAACGCCCTCTT‑‑AGAGAGCGTCACTAAGTTCCACTGCGGGGTTATCTACGactact                                                                                                                                                                                                                          >  1:340235‑M2/209‑301 (MQ=255)`  
                                                                                 `tgattaagaagttaggccaactgctggttcgtatgtacaacgtggaagccaagcgactgaacgatgaggctcgtaaagaggccacacagtcacgcgctctggcgattcgctccaacgaactggctgacagtgcatccactaaagttaccgaggctgcccgtgtggcaaaccaagctcaacagctttccaaattctttgagtaatcaaTAGGAGAAATCAATATGATCGTTTCTGACATCGAAGCTAACGCCCTCTT‑‑AGAGAGCGTCACTAAGTTCCACTGCGGGGTTATCTACGACTACTc                                                                                                                                                                                                                         <  1:406597‑M2/94‑1 (MQ=255)`  
                                                                                  `gattaagaagttaggccaactgctggttcgtatgtacaacgtggaagccaagcgcctgtacgatgaggctcgtaacgaggccacacagtcacgcgctctggcgattagctccaacgaactggctgacagtgcatccactcaagttaccgaggctgcccgtgtggcaaaccaagctcaacagctttccaaattcttttcgtaatcaaTAGTAGAAATCCATATGATCGTTTCTGACATCTAAGCTACCGCCCTCTT‑‑AGCGAGCGTCACTAAGTTCCACTGCGGGGTTTTatacgactcttcc                                                                                                                                                                                                                        >  1:322025‑M2/207‑296 (MQ=255)`  
                                                                                  `attaagaagttaggccaactgctggttcgtatgtacaacgtggaagccaagcgactgaacgatgaggctcgtaaagaggccacacagtcacgcgctctggcgattcgctccaacgaactggctgacagtgcatccactaaagttaccgaggctgcccgtgtggcaaaccaagctcaacagctttccaaattctttgagtaatccaaCAGGAGAAATCAATATGATCGTTTCTGACATCGAAGCTAACTCCCTCTT‑‑AGAGAGCGTCACTCAGTTCCACTGCGGGGTTATCTACGACTACTcc                                                                                                                                                                                                                        >  1:387305‑M2/207‑301 (MQ=255)`  
                                                                                    `taagaagttaggcaaactgctggttcgtatgtacaacgtggaagccaagcgaatgaatgatgaggctcgtaaagaggccacacagtcacgcgctctggcgattcgctccaacgaactggctgacagtgcatccactaaagttaccgaggctgcccgtgtggcaaaccaagctcaacagctttccaaattctttgagtaatcaaaCAGGAGAAATCAATATGATCGTTTCTGACATCGAAGCTAACGCCCTCTT‑‑AGAGAGCGTCACTAAGTTCCACTGCGGGGTTATCTACGACTACTCCAc                                                                                                                                                                                                                      <  1:507351‑M2/97‑1 (MQ=255)`  
                                                                                       `cggagatgtgtataagagacagcgtatgtacaacgtggaagccaagcgactgaacgatgaggctcgtaaagaggccacacagtcacgcgctctggcgattcgctccaacgaactggctgacagtgcatccactaaagttaccgaggctgcccgtgtggcaaaccaagctcaacagctttccaaattctttgagtaatcaaaCAGGAGAAATCAATATGATCGTTTCTGACATCGAAGCTAACGCCCTCTT‑‑AGAGAGCGTCACTAAGTTCCACTGCGGGGTTATCTACGACTACTCCACCGc                                                                                                                                                                                                                   <  1:172679‑M2/100‑1 (MQ=255)`  
                                                                                        `gaagttaggccaactgctggttcgtatgtacaacgtggaagccaagcgactgaacgatgaggctcgtaaagaggccacacagtcacgcgctctggcgattcgctccaacgaactggctgacagtgcatccactaaagttaccgaggctgcccgtgtggcaaaccaagctcaacagctttccaaattctttgagtaatcaaTAGGAGAAATCAATATGATCGTTTCTGACATCGAAGCTAACGCCCTCTT‑‑AGAGAGCGTCACTAAGTTCCACTGCGGGGTTATCTACGCCTACTCCACCGCt                                                                                                                                                                                                                  >  1:18978‑M2/201‑301 (MQ=255)`  
                                                                                          `agttaggccaactgctggttcgtatgtacaacgtggaagccaagcgactgaacgatgaggctcgtaaagaggccacacagtcacgcgctctggcgattcgctccaacgaactggctgacagtgcatccactaaagttaccgaggctgcccgtgtggcaaaccaagctcaacagctttccaaattctttgagtaatcaaTAGGAGAAATCAATATGATCGTTTCTGACATCGAAGCTAACGCCCTCTT‑‑AGAGAGCGTCACTAAGTTCCACTGCGGGGTTATCTACGACTActccacctgtct                                                                                                                                                                                                                >  1:471938‑M2/199‑296 (MQ=255)`  
                                                                                          `agttaggccaactgctggttcgtatgtacaacgtggaagccaagcgactgaacgatgaggctcgtaaagaggccacacagtcacgcgctctggcgattcgctccaacgaactggctgacagtgcatccactaaagttaccgaggctgcccgtgtggcaaaccaagctcaacagctttccaaattctttgagtaatcaaTAGGAGAAATCAATATGATCGTTTCTGACATCGAAGCTAACGCCCTCTT‑‑AGAGAGCGTCACTAAGTTCCACTGCGGGGTTATCTACGACTACTCCACCGCTGa                                                                                                                                                                                                                >  1:529133‑M2/199‑301 (MQ=255)`  
                                                                                           `gttaggccaactgctggtgcgtacgtacaacgtggaagccaagcgactgaacgctgaggctcgtaaagaggccacacagtcacgcgctctggcgattcgctccaacgaactggctgacagtgcatccactaaagttaccgagtctgcccgtgtggcaaaccaagctcaacagctttccaaattctttgagtaatcaaTAGGAGAAATCAATATGATCGTTTCTGACATCGAAGCTAACGCCCTCTT‑‑AGAGAGCGTCACTAAGTTCCACTGCGGGGTTATCTACGACTACTCCACCGCTGAg                                                                                                                                                                                                               <  1:419214‑M2/104‑1 (MQ=255)`  
                                                                                               `ggccaactgctggttcgtatgtacaacgtggaagccaagcgactgaacgatgaggctcgtaaagaggccacacagtcacgcgctctggcgattcgctccaacgaactggctgacagtgcatccactaaagttaccgaggctgcccgtgtggcaaaccaagctcaacagctttccaaattctttgagtaatcaaTAGGAGAAATCAATATGATCGTTTCTGACATCGAAGCTAACGCCCTCTT‑‑AGAGAGCGTCACTAAGTTCCACTGCGGGGTTATCTACGACTActccaccgcctgtctct                                                                                                                                                                                                           >  1:400281‑M2/194‑293 (MQ=255)`  
                                                                                               `ggccaactgctggttcgtatgtacaacgtggaagccaagcgactgaacgatgaggctcgtaaagaggccacacagtcacgcgctctggcgattcgctccaacgaactggctgacagtgcatccactaaagttaccgaggctgcccgtgtggcaaaccaagctcaacagctttccaaattctttgagtaatcaaTAGGAGAAATCAATATGATCGTTTCTGACATCGAAGCTAACGCCCTCTT‑‑AGAGAGCGTCACTAAGTTCCACTGCGGGGTTATCTACGACTACTCCACCGCTGAGTACg                                                                                                                                                                                                           <  1:363359‑M2/108‑1 (MQ=255)`  
                                                                                                 `ccaactgctggttcgtatgtacaacgtggaagccaagcgactgaacgatgaggctcgtaaagaggccacacagtcacgcgctctggcgattcgctccaacgaactggctgacagtgcatccactaaagttaccgaggctgcccgtgtggcaaaccaagctcaacagctttccaaattctttgagtaatcaaTAGGAGAAATCAATATGATCGTTTCTGACATCGAAGCTAACGCCCTCTT‑‑AGAGAGCGTCACTAAGTTCCACTGCGGGGTTATCTACGACTACTCCACCGCTGAGTACGTa                                                                                                                                                                                                         <  1:596038‑M2/110‑1 (MQ=255)`  
                                                                                                         `ggttcgtatgtacaacgtggaagccaagcgactgaacgatgaggctcgtaaagaggccacacagtcacgcgctctggcgattcgctccaacgaactggctgacagtgcatccactaaagttaccgaggctgcccgtgtggcaaaccaagctcaacagctttccaaattctttgagtaatcaaaCAGGAGAAATCAATATGATCGTTTCTGACATCGAAGCTAACGCCCTCTTAGAGAGAGCGTCACTAAGTTCCACTGCGGGGTTATCTACGACTACTCCACCGCTGAGTACGTAAgctgt                                                                                                                                                                                                   >  1:267395‑M2/184‑299 (MQ=255)`  
                                                                                                         `gagacagatgtacaacgtggaagccaagcgactgaacgatgaggctcgtaaagaggccacacagtcacgcgctctggcgattcgctccaacgaactggatgacagtgcatccactaaagttaccgaggctgcccgtgtggcaaaccaagctcaacagctttccaaattctttgagtaatcaaaCAGGAGAAACCATTATGATCGTTTCTGACATCGAAGCTAACGCCCTCTT‑‑AGAGAGCGTCACTAAGTTCCACTGCGGGGTTATCTACGACTACTCCACCGCTGAGTACGTAAGCTACCg                                                                                                                                                                                                 <  1:292728‑M2/118‑1 (MQ=255)`  
                                                                                                              `tgtatgtacaacgtggaagccaagcgactgaacgatgaggctcgtaaagaggccacacagtcacgcgctctggcgattcgctccaacgaactggctgacagtgcatccactaaagttaccgaggctgcccgtgtggcaaaccaagctcaacagctttccaaattctttgagtaatcaaTAGGAGAAATCAATATGATCGTTTCTGACATCGAAGCTAACGCCCTCTT‑‑AGAGAGCGTCACTAAGTTCCACTGCGGGGTTATCTACGACTACTCCACCGCTGAGTACGTAAGCTACCgtcctg                                                                                                                                                                                            >  1:549845‑M2/179‑299 (MQ=255)`  
                                                                                                              `cgtatgtacaacgtggaagccaagcgactgaacgatgaggctcgtaaagaggccacacagtcacgcgctctggcgattcgctccaacgaactggctgacagtgcatccactaaagttaccgaggctgcccgtgtggcaaaccaagctcaacagctttccaaattctttgagtaatcaaTAGGAGAAATCAATATGATCGTTTCTGACATCGAAGCTAACGCCCTCTT‑‑AGAGAGCGTCACTAAGTTCCGCTGCGGGGTTATCTACGACTACTCCACCGCTGAGTACGTAAGCTACCgtcctg                                                                                                                                                                                            >  1:334153‑M2/179‑299 (MQ=255)`  
                                                                                                                 `gagacagacgtggaagccaagcgactgaacgatgaggctcgtaaagaggccacacagtcacgcgctctggcgattcgctccaacgaactggctgacagtgcatccactaaagttaccgaggctgcccgtgtggcaaaccaagctcaacagctttccaaattctttgagtaatcaaTAGGAGAAATCAATATGATCGTTTCTGACATCGAAGCTAACGCCCTCTT‑‑AGAGAGCGTCACTAAGTTCCACTGCGGGGTTATCTACGACTACTCCACCGCTGAGTACGTAAGCTACCGTCCGAGTg                                                                                                                                                                                         <  1:635454‑M2/126‑1 (MQ=255)`  
                                                                                                                   `gtaaaacgtggaaaccaagcgactgaactatgaggctcgtaaagaggccacacagtcacgcgctctggcgattcgctccaacgaactggctgacagtgcatccaataaagttaccgaggctgcccgtggggcaaaccaagctcaacagctttccaaattctttgagtaatcaaTAGGAGAAATCAATATGATCGTTTCTGACATCGAAGCTAACGCCCTCTT‑‑AGAGAGCGTCACTAAGTTCCACTGCGGGGTTATCTACGACTACTCCACCGCTGAGTACGTAAGCTACCGTCCGAGTGAc                                                                                                                                                                                       <  1:243804‑M2/128‑1 (MQ=255)`  
                                                                                                                     `aaacgtggaagccaagcgactgaacgatgaggctcgtaaagaggccacacagtcacgcgctctggcgattcgctccaacgaactggctgacagtgcatccactaaagttaccgaggctgcccgtgtggcaaaccaagctcaacagctttccaaattctttgagtaatcaaaCAGGAGAAATCAATATGATCGTTTCTGACATCGAAGCTAACGCCCTCTT‑‑AGAGAGCGTCACTAAGTTCCACTGCGGGGTTATCTACGACTACTCCACCGCTGAGTACGTAAGCTACCGTCCGAGTGACtt                                                                                                                                                                                     <  1:130196‑M2/130‑1 (MQ=255)`  
                                                                                                                      `aaacgtggaagccaagcgactgaacgatgaggctcgtaaagagtccacacagtcacgcgctatggcgattcgctccaacgaactggctgacagtgcatccactaaagttaccgaggctgcccgtgtggcaaaccaagctcaacagctttccaaattctttgagtaatcaaTAGGAGAAATTAAGATGATCGTTTCTGACATCGAAGCTAACGCCCTCTT‑‑AGAGAGCGTCACTAAGTTCCACTGCGGGGTTATCTACGACTACTCCACCGCTGAGTACGTAAGCTACCGTCCGAGTGACTTc                                                                                                                                                                                    <  1:75927‑M2/131‑1 (MQ=255)`  
                                                                                                                         `gtggaagccaagcgactgaacgatgaggctcgtaaagaggccacacagtcacgcgctctggcgattcgctccaacgaactggctgacagtgcatccactaaagttaccgaggctgcccgtgtggcaaaccaagctcaacagctttccaaattctttgagtaatcaaaCAGGAGAAATCAATATGATCGTTTCTGACATCGAAGCTAACGCCCTCTT‑‑AGAGAGCGTCACTAAGTTCCACTGCGGGGTTATCTACGACTACTCCACCGCTGAGTACGTAAGCTACCGTCCGAGTGACTTCGGt                                                                                                                                                                                 >  1:159920‑M2/168‑301 (MQ=255)`  
                                                                                                                                `gccaagcgactgaacgatgaggctcgtaaagaggccacacagtcacgcgctctggcgattcgctccaacgaactggctgacagtgcatccactaaagttaccgaggctgcccgtgtggcaaaccaagctcaacagctttccaaattctttgagtaatcaaTAGGAGAAATCAATATGATCGTTTCTGACATCGAAGCTAACGCCCTCTT‑‑AGAGAGCGTCACTAAGTTCCACTGCGGGGTTATCTACGACTACTCCACCGCTGAGTACGTAAGCTACCGTCCGAGTGACTTCGGTGCGTATc                                                                                                                                                                          <  1:286481‑M2/141‑1 (MQ=255)`  
                                                                                                                                `ccaagcgactgaacgatgaggctcgtaaagaggccacacagtcacgcgctctggcgattcgctccaacgaactggctgacagtgcatccactaaagttaccgaggctgcccgtgtggcaaaccaagctcaacagctttccaaattctttgagtaatcaaaCAGGAGAAATCAATATGATCGTTTCTGACATCGAAGCTAACGCCCTCTT‑‑AGAGAGCGTCACTAAGTTCCACTGCGGGGTTATCTACGACTACTCCACCGCTGAGTACGTAAGCTACCGTCCGAGTGACTTCGGTGCGTATc                                                                                                                                                                          <  1:568408‑M2/141‑1 (MQ=255)`  
                                                                                                                                  `caagcgactgaacgatgaggctcgtaaagaggccacacagtcacgcgctctggcgattcgctccaacgaactggctgacagtgcatccactaaagttaccgaggctgcccgtgtggcaaaccaagctcaacagctttccaaattctttgagtaatcaaTAGGAGAAATCAATATGATCGTTTCTGACATCGAAGCTAACGCCCTCTT‑‑AGAGAGCGTCACTAAGTTCCACTGCGGGGTTATCTACGACTACTCCACCGCTGAGTACGTAAGCTACCGTCCGAGTGACTTCGGTGCGTATCTg                                                                                                                                                                        <  1:201953‑M2/143‑1 (MQ=255)`  
                                                                                                                                  `aagcgcgtgaacgatgaggctcgtaaagaggccacacagtcacgcgctctggcgattcgctccaacgaactggctgacagtgcatccactaaagttaccgaggctgcccgtgtggcaaaccaagctcaacagctttccaaattctttgagtaatcaaaCAGGAGAAACCATTATGATCGTTTCTGACATCGAAGCTAACGCCCTCTT‑‑AGAGAGCGTCACTAAGTTCCACTGCGGGGTTATCTACGACTACTCCACCGCTGAGTACGTAAGCTACCGTCCGAGTGACTTCGGTGCGTATCTg                                                                                                                                                                        <  1:435927‑M2/143‑1 (MQ=255)`  
                                                                                                                                    `agcgactgaacgatgaggctcgtaaagaggccacacagtcacgcgctctggcgattcgctccaacgaactggctgacagtgcatccactaaagttaccgaggctgcccgtgtggcaaaccaagctcaacagctttccaaattctttgagtaatcaaTAGGAGAAATCAATATGATCGTTTCTGACATCGAAGCTAACGCCCTCTT‑‑AGAGAGCGTCACTAAGTTCCACTGCGGGGTTATCTACGACTACTCCACCGCTGAGTACGTAAGCTACCGTCCGAGTGACTTCGGTGCGTATCTGGa                                                                                                                                                                      <  1:535432‑M2/145‑1 (MQ=255)`  
                                                                                                                                                    `gctcgtaaagaggccacacagtcacgcgctctggcgattcgctccaacgaactggctgacagtgcatccactaaagttaccgaggctgcccgtgttgcaaaccaagctcaacagctttccaaattctttgagtaatcaaaCAGGAGAAACCATTATGATCGTTTCTGCCATCGAAGCTAACGCCCTCTT‑‑AGAGAGCGTCACTAAGTTCCACTGCGGGGTTATCTACGACTACTCCCCCGCTGAGTACGTAAGCTACCGTCCGAGTGACttcggtgcgtatctggactgtctcttatacccc                                                                                                                                                      >  1:255145‑M2/141‑285 (MQ=255)`  
                                                                                                                                                            `aagagtccacacagtcacgcgctctggcgattcgctccaacgaactggctgacagtgcatccactaaagttaccgaggctgcccgtgtggcaaaccaagctcaacagctttccaaattctttgagtaatcaaTAGGAGAAATCAATATGATCGTTTCTGACATCGAAGCTAACGCCCTCTT‑‑AGAGAGCGTCACTAAGTTCCACTGCGGGGTTATCTACGACTACTCCACCGCTGAGTACGTAAGCTACCGTCCGAGTGACTTCGGTGCGTATCTGGATGCGCTGGAAGCCGAGGTTGCACg                                                                                                                                              <  1:590142‑M2/169‑1 (MQ=255)`  
                                                                                                                                                               `aggccacacagtcacgcgctctggcgattcgctcctaagaactggctgacagtgcaaccactaaagttaccgaggctgcccgtgtggcaaaccatgctcaacagctttccaaattctttgagtaatcaaTATGATAAATCAATATGATCGTTTCTGACATCGAAGCTAACGCCCTCTT‑‑AGAGAGCGTCACTAAGTTCCACTGCGGGGATATCTACGACTACTCCACCGCTGTGTACGTAAGCTACCGTCCGAATGACTTAGGTGCGTACCTGGATACGCTGGAAGCCGGAGTTGCACGAgg                                                                                                                                           >  1:105435‑M2/130‑301 (MQ=255)`  
                                                                                                                                                                `ggccacacaaccacgcgctctggcgattcgctccaacgaactggctgacagtgcatccactaaagttaccgaggctgcccgtgtggcaaaccaagctcaacagctttccaaattctttgagtaatcaaTAGGAGAAATCAATATGATCGTTTCCGACATCGAAGCTAACGCCCTCTT‑‑AGAGAGCGTCACTAAGTTCCACTGCGGGGTTATCTACGACTACTCCACCGCTGAGTACGTAAGCTACCGTCCGAGTGACTTCGGTGCGTATCTGGATGCGCTGGAAGCCGAGGTTGCACGAGGc                                                                                                                                          <  1:123537‑M2/173‑1 (MQ=255)`  
                                                                                                                                                                         `gtcacgcgctctggcgattcgctccaacgaactggctgacagtgcatccactaaagttaccgaggctgcccgtgtggcaaaccaagctcaacagctttccaaattctttgagtaatcaaTAGGAGAAATCAATATGATCGTTTCTGACATCGAAGCTAACGCCCTCTT‑‑AGAGAGCGTCACTAAGTTCCACTGCGGGGTTATCTACGACTACTCCACCGCTGAGTACGTAAGCTACCGTCCGAGTGACTTCGGTGCGTAACTGGATGCGCTGGAAGCCGAGGTTGCACGAGGCGGTCTTAtt                                                                                                                                 >  1:394924‑M2/120‑301 (MQ=255)`  
                                                                                                                                                                          `tcccgcgctctggcaattcgttccaacgaactggctgacagtgcatccactaaagttaccgaggctgcccgtgtggcaaaccaagctcaacagctttccaaattctttgagtaatcaaTAGGAGAAATCAATATGATCGTTTCTGACATCGAAGCTAACGCCCTCTT‑‑AGAGAGCGTCACTAAGTTCCACTGCGGGGTTATCTACGACTACTCCACCGCTGAGTACGTAAGCTACCGTCCGAGTGACTTCGGTGCGTATCTGGATGCGCTGGAAGCCGAGGTTGCACGAGGCGGTCTTATtg                                                                                                                                <  1:641278‑M2/183‑1 (MQ=255)`  
                                                                                                                                                                          `gcacgcgctctggcgattcgctccaacgaactggctgacagtgcatcaactaaagttaccgaggctgaccgtgtggaaaaccaagctcaacagctttccaaattctttgagtaatcaaTAGGAGAAAGCAATATGATCGTTTCTGACATCGAAGCTAACGCCCTCTT‑‑AGAGAGCGTCACTAAGTTCCACTGCGGGGTTATCTACGACTACTCCACCGCTGAGTACGTAAGCTACCGTCCGAGTGACTTCGGTGCGTATCTGGATGCGCTGGAAGCCGAGGTTGCACGAGGCGGTCTTATtg                                                                                                                                <  1:184516‑M2/183‑1 (MQ=255)`  
                                                                                                                                                                          `cgggggctcggagatgtgtataagagacagctggctgacagtgcatccactaaagttaccgaggctgcccgtgtggcaaaccaagctcaacagctttccaaattctttgagtaatcaaTAGGAGAAATCAATATGATCGTTTCTGACATCGAAGCTAACGCCCTCTT‑‑AGAGAGCGTCACTAAGTTCCACTGCGGGGTTATCTACGACTACTCCACCGCTGAGTACGTAAGCTACCGTCCGAGTGACTTCGGTGCGTATCTGGATGCGCTGGAAGCCGAGGTTGCACGAGGCGGTCTTATtg                                                                                                                                <  1:424970‑M2/183‑1 (MQ=255)`  
                                                                                                                                                                          `cacgcgctctggcgattcgctccaacgaactggctgacagtgcatccactaaagttaccgaggctgcccgtgttgcaaacctagctcaacagctttccaaattctttgagtaatcaaaCAGGAGAAATCAATATGATCTTTTCTGACATCAAAGCTAACGCCCTCTT‑‑AGAGAGCGTCACTAAGTTCCACTTCGGGGTTATCTACGACTACTCCACCGCTGAGTACGTAAGCTACCGTCCGAGTGACTTCGGTGCGTATCTGGATGCGCTGGAAGCCGAGGTTGCACGAGGCGGTCTTATtg                                                                                                                                <  1:482687‑M2/183‑1 (MQ=255)`  
                                                                                                                                                                          `cacgcgctctggcgattcgctccaacgaactggctgacagtgcatccactaaagttaccgaggctgcccgtgtggcaaaccaagctcaacagctttccaaattctttgagtaatcaaaCAGGAGAAATCAATATGATCGTTTCTGACATCGAAGCTAACGCCCTCTT‑‑AGAGAGCGTCACTAAGTTCCACTGCGGGGTTATCTACGACTACTCCACCGCTGAGTACGTAAGCTACCGTCCGAGTGACTTCGGTGCGTATCTGGATGCGCTGGAAGCCGAGGTTGCACGAGGCGGTCTTATtg                                                                                                                                <  1:523842‑M2/183‑1 (MQ=255)`  
                                                                                                                                                                          `cacgcgctcgggcgattcgctccaacgaactggctgacagtgcatccactaaagttaccgaggatgcccgtgtggcaaaccaagctcaacagctttccaaatgctttgagtaatcaaaCAGGAGAAATCAATATGATCGTGTCTGACATCGAAGCTAACGCCCTCTT‑‑AGAGAGCGTCACTAAGTTCCACTGCGGGGTTAGCTACGACTACTCCACCGCTGAGTACGTAAGCTACCGTCCGAGTGACTTCGGTGCGTATCTGGATGCGCTGGAAGCCGAGGTTGCACGAGGCGGTCTTATtg                                                                                                                                <  1:430183‑M2/183‑1 (MQ=255)`  
                                                                                                                                                                          `aacgcgctagggagattcgatccaacaaaccggcagaccgtgaatctacgaaagttaacgaggctgcccgtgtggcaaaccaagcgcaacagctttccaaagtctgtgagtaatcaaaCAGGAGAAATCAAAATGATCGTTTCTGACATGGAAGCTAACCACCTCGT‑‑AGAGAGCGTCACTAAGTTCCACTGCGGGGTTATCTACGACTACTCCACCGGTGAGTACGTAAGCTACCGTCCGAGTGACTTCGGTGCGTCTCTGGATGCGCTGGAAGCCGAGGTTGCACGAGGCGGTATTATtg                                                                                                                                <  1:500080‑M2/183‑1 (MQ=255)`  
                                                                                                                                                                                   `ctggcgattcgctccaacgaactggctgacagtgcatccactaaagttaccgaggctgcccgtgtggcaaaccaagctcaacagctttccaaattctttgagtaatcaaTTGGAGAAATCAATATGATCGTTTCTGACATCGAAGCTAACGCCCTCTT‑‑AGAGAGCGTCACTAAGTTCCACTGCGGGGTTATCTACGACTACTCCACCGCTGAGTACGTAAGCTACCGTCCGAGTGACTTCGGTGCGTATCTGGATGCGCTGGAAGCCGAGGTTGCACGAGGcggtcttattgctgtctctt                                                                                                                       >  1:465822‑M2/110‑292 (MQ=255)`  
                                                                                                                                                                                               `ccaacgaactggctgacagtgcatccactaaagttaccgaggctgcccgtgtggcaaaccaagctcaacagctttccaaattctttgagtaatcaaaCAGGAGAAACCATTATGATCGTTTCTGACATCGAAGCTAACGCCCTCTT‑‑AGAGGGCGTCACTAAGTTCCACTGCGGGGTTATATACGACTACTCCACCGCTGAGTACGTAAGCTACCGTCCGAGTGACTTCGGTGCGTATCTGGATGCGCTGGAAGCCGAGGTTGCACGAGGCGGTCTTATTGTTTTCCACAACGGTCACAagc                                                                                                           >  1:80891‑M2/98‑300 (MQ=255)`  
                                                                                                                                                                                                   `ggaactggctgaaagtgcatacactaaagttaccgaggctgcccgtgtggcaaaccaagctcaacagctttccaaagtctttgagtaatcaaaCAGGAGAAATCAATATGATCGTTTCTGACATCGAAGCTAACGCCCTCTT‑‑AGAGAGCGTCACTAAGTTCCACTGCGGGGTTATCTACGACTACTCCACCGCTGAGTACGTAAGCTACCGTCCGAGTGACTTCGGTGCGTATCTGGATGCGCTGGAAGCCGAGGTTGCACGAGGCGGTCTTATTGTGTTCCACAACGGTCACAAGTATGa                                                                                                       <  1:310033‑M2/208‑1 (MQ=255)`  
                                                                                                                                                                                                   `acgaactggctgacagtgcatccactaaagttaccgaggctgcccgtgttgcaaaccaagctcaacagctttccaaattctttgagtaatcaaTAGGAGAAATCAATATGATCGTTTCTGACATCGAAGCTAACGCCCTCTT‑‑AGAGAGCGTCACTAAGTTCCACTGCGGGGTTATCTACGACTACTCCACCGCTGAGTACGTAAGCTACCGTCCGAGTGACTTCGGTGCGTATCTGGATGCGCTGGAAGCCGAGGTTGCACGAGGCGGTCTTATTGTGTTCCACAACGGTCACAAGTATGa                                                                                                       <  1:476779‑M2/208‑1 (MQ=255)`  
                                                                                                                                                                                                              `gacagtgcatccactaaagttaccgaggctgcccgtgtggcaaaccaagctcaacagctttccaaattctttgagtaatcaaTAGGAGAAATCAATATGATCGTTTCTGACATCGAAGCTAACGCCCTCTT‑‑AGAGAGCGTCACTAAGTTCCACTGCGGGGTTATCTACGACTACTCCACCGCTGAGTACGTAAGCTACCGTCCGAGTGACTTCGGTGCGTATCTGGATGCGCTGGAAGCCGAGGTTGCACGAGGCGGTCTTATTGTGTTCCACAACGGTCACAAGTATGACGTTCCTGCAt                                                                                            <  1:304367‑M2/219‑1 (MQ=255)`  
                                                                                                                                                                                                                        `ccactaaagttaccgaggctgcccgtgtggcaaaccaagctcaacagctttccaaattctttgagtaatcaaTAGGAGAAATCAATATGATCGTTTCTGACATCGAAGCTAACGCCCTCTT‑‑AGAGAGCGTCACTAAGTTCCACTGCGGGGTTATCTACGACTACTCCACCGCTGAGTACGTAAGCTACCGTCCGAGTGACTTCGGTGCGTATCTGGCTGCGTTGGAAGCCGAGGTTGCACGAGGAGGTCTTATTGTGTTCCACAACGGTCACAAGTATGACGTTCCTGCATTGCCCAACCt                                                                                  >  1:454577‑M2/73‑301 (MQ=255)`  
                                                                                                                                                                                                                        `ccactaaagttaccgaggctgcccgtgtggcaaaccaagctcaacagctttccaaattctttgagtaatcaaTAGGAGAAATCAATATGATCGTTTCTGACATCGAAGCTAACGCCCTCTT‑‑AGAGAGCGTCACTAAGTTCCACTGCGGGGTTATCTACGACTACTCCACCGCTGAGTACGTAAGCTACCGTCCGAGTGACTTCGGTGCGTATCTGGATGCGCTGGAAGCCGAGGTTGCACGAGGCGGTCTTATTGTGTTCCAcaacggtcacaagtatgacgctgtctcttatacacatct                                                                                  >  1:249112‑M2/73‑282 (MQ=255)`  
                                                                                                                                                                                                                              `aagttaccgaggctgcccgtgtggcaaaccaagctcaacagctttccaaattctttgagtaatcaaTAGGAGAAATCAATATGATCGTTTCTGACATCGAAGCTAACGCCCTCTT‑‑AGAGAGCGTCACTAAGTTCCACTGCGGGGTTATCTACGACTACTCCACCGCTGAGTACGTAAGCTACCGTCCGAGTGACTTCGGTGCGGATCTGGAGGCGCTGGAAGCCGAGGTTGCACGAGGCGGTCTTATTGGGTTCCACAACGGTCACAAGTATGACGTGCCTGACTTGACAAAACGGGaaaa                                                                            >  1:315013‑M2/67‑301 (MQ=255)`  
                                                                                                                                                                                                                               `agttaccgaggctgcccgtgtggcaaaccaagctcaacagctttccaaattctttgagtaatcaaTAGGAGAAATCAATATGATCGTTTCTGACATCGAAGCTAACGCCCTCTT‑‑AGAGAGCGTCACTAAGTTCCACTGCGGGGTTATCTACGACTACTCCACCGCTGAGTACGTAAGCTACCGTCCGAGTGACTTCGGTGCGTATCTGGATGCGCTGGAAGCCGAGGTTGCACGAGGCGGTCTTATTGTGTTCCACAACGGTCACAAGTATGACGTTCCTGCATTGACCAAACTGGCAAAg                                                                           >  1:601114‑M2/66‑301 (MQ=255)`  
                                                                                                                                                                                                                                     `gaggctgcccgtgtggcaaaccaagctcaacagctttccaaattctttgagtaatcaaaCAGGAGAAACCATTATGATCGTTTCTGACATCGAAGCTAACGCCCTCTT‑‑AGAGAGCGTCACTAAGTTCCACTGCGGGGTTATCTACGACTACTCCACCGCTGAGTACGTAAGCTACCGTCCGAGTGACTTCGGTGCGTATCTGGATGCGCTGGAAGCCGAGGTTGCACGAGGCGGTCTTATTGTGTTCCACAACGGTCACAAGTATGACTTTCCTGCATTGACAAAACTGGCAAAGTTGCaa                                                                     >  1:5557‑M2/60‑301 (MQ=255)`  
                                                                                                                                                                                                                                                              `gctcaacagctttccaaattctttgagtaatcaaTAGGAGAAATCAATATGATCGTTTCTGACATCGAAGCTAACGCCCTCTT‑‑AGAGAGCGTCACTAAGTTCCACTGCGGGGTTATCTACGACTACTCCACCGCTGAGTACGTAAGCTACCGTCCGAGTGACTTCGGTGCGTATCTGGATGCGCTGGAAGCCGAGGTTGCACGAGGCGGTCTTATTGTGTTCCACAACGGTCACAAGTATGACGTTCCTGCATTGACCAAACTGGCAAAGTTGCAATTGAACCGAGAGTTCCACCTTCCTc                                            >  1:630653‑M2/35‑301 (MQ=255)`  
                                                                                                                                                                                                                                                                         `tccaaattctttgagtaatcaaacaGGAGAAATCAATATGATCGTTTCTGACATCGAAGCTAACGCCCTCTT‑‑AGAAAGCGTAACTAATTTCAACTGCGGGGTTATCTACGACTCCTCCACGGCTAAGTAGTTAGGCTACCGTCGAGGTACCTTCGTTGGGTATCGGGATGCGCTGGAAGCCGAGGTTGCACGAGGCGGCCTTATTGTGTTCACAAACGGTCACAAGTATGACGTCCCTGCTTTGACCAAACGGGCAAAGTTGCAATTGAACGGAGAGTTCAACCTTCCTCTTGAGACCttt                                 >  1:458683/25‑299 (MQ=255)`  
                                                                                                                                                                                                                                                                         `tccaaattctctgagtaatcaaaCAGGAGAAATCAATATGATCGTTTCTGACATCGAAGCTAACGCCCTCTT‑‑AGAGAGCGTCACTAAGTTCCACTGCGGGGTTATCTACGACTACTCCACCGCTGAGTACGTAAGCTACCGTCCGAGTGACTTCGGTGCGTATCTGGATGCGCTTGAAGCCGAGGTTGCACGAGGCGGTCTTATTGTGTTCCACAACGGTCACAAGTATGACGTTACTGCATTGACCAAACTGGCCAAGTTGCAATTGAACCGAGCGTTCCACCTTCCTCGTGAGAACTGt                                 >  1:327400‑M2/24‑301 (MQ=255)`  
                                                                                                                                                                                                                                                                              `aattctttgagtaatcaaTAGGAGAAATCAATATGATCGTTTCTGACATCGAAGCTAACGCCCTCTT‑‑AGAGAGCGTCACTAAGTTCCACTGCGTGGTTATCTACGACTACTCCACCGCTGAGTACGTAAGCTACCGTCCGAGTGACTTCGGTGCGTATCTGGATGCGCTGGAAGCCGAGGTTGCACGAGGCGGTCTTATTGTGTTCCACAACGGTCACAAGTATGACGTTCCTGCATTGACCAAACTGGCAAAGTTGCAATTGAACCGAGAGTTCCACCTTCCTCGTGagaactgctgtct                            >  1:230942‑M2/19‑295 (MQ=255)`  
                                                                                                                                                                                                                                                                              `aattctttgagtaatcaaTAGGAGAAATCAATATGATCGTTTCTGACATCGAAGCTAACGCCCTCTT‑‑AGAGAGCGTCACTAAGTTCCACTGCGGGGTTATCTACGACTACTCCACCGCTGAGTACGGAAGCTACCGTCCGAGTGACTTCGGTGCGTATCTGGATGCGCTGGAAGCCGAGGTTGCACGAGGCGGTCTTATTGTGTTCCACAACGGTCACAAGTATGACGTTCCTGCATTGACCAAACTGGCAAAGTTGCAATTGAACCGAGAGTTCCACCTTCCTCGTGAGAACTGTATTGa                            <  1:625022‑M2/283‑1 (MQ=255)`  
                                                                                                                                                                                                                                                                               `attctttgagtaatcaaTAGGAGAAATCAATATGATCGTTTCTGACATCGAAGCTAACGCCCTCTT‑‑AGAGAGCGTCACTAAGTTCCACTGCGGGGTTATCTACGACTACTCCACCGCTGAGTACGTAAGCTACCGTCCGAGTGACTTCGGTGCGTATCTGGATGCGCTGGAAGCCGAGGTTGCACGAGGCGGTCTTATTGTGTTCCACAACGGTCACAAGTATGACGTTCCTGCATTGACCAAACTGGCAAAGTTGCAATTGAACCGAGAGTTCCACCTTCCTCGTGAGAACTGTATTGac                           <  1:667196‑M2/284‑1 (MQ=255)`  
                                                                                                                                                                                                                                                                                `ttcgttgattaatcaaTCGGAGAAATCAATTTGGTCGTTTCTGACAACGAAGCTAACCCCCTCTT‑‑AGCGAGCGTCACTAAGTTCCACTGCGGGGTTATCTACGCCTACTCCACCGCTGAGTACGTAAGCTACCGTCCGAGTGACTTTGGTGCGTATCTGGATGTGCTGGAAGCCGAGGTTGCACGAGGCGGTCTTATTGTGTTCCACAACGGTCACAAGTATGACGTTCCTGCATTGACCAAACTGGCAAAGTTGCAATTGAACCGAGAGTTCCACCTTCCTCGTGAGTACTGTATTGaca                          <  1:297868‑M2/285‑1 (MQ=255)`  
                                                                                                                                                                                                                                                                                         `aagagacagggAGAAATCAATTTGATCGTTTCTGACATCGAAGCTAACGCCCTCTT‑‑AGAGAGCGTCACTAAGTTCCACTGCGGGGTTATCTACGACTACTCCACCGCTGAGTACGTAAGCTACCGTCCGAGTGACTTCGGTGCGTATCTGGATGCGCTGGAAGCCGAGGTTGCACGAGGCGGTCTTATTGTGTTCCACAACGGTCACAAGTATGACGTTCCTGCATTGACCAAACTGGCAAAGTTGCAATTGAACCGAGAGTTCCACCTTCCTCGTGAGAACTGTATTGACACCCTTgtgt                 <  1:135985/292‑1 (MQ=255)`  
                                                                                                                                                                                                                                                                                         `aagagacagggAGAAATCAATATGATCGTTTCTGACATCGAAGCTAACGCCCTCTT‑‑AGAGAGCGTCACTAAGTTCCACTGCGGGGTTATCTACGACTACTCCACCGCTGAGTACGTAAGCTACCGTCCGAGTGACTTCGGTGCGTATCTGGATGCGCTGGAAGCCGAGGTTGCACGAGGCGGTCTTATTGTGTTCCACAACGGTCACAAGTATGACGTTCCTGCATTGACCAAACTGGCAAAGTTGCAATTGAACCGAGAGTTCCACCTTCCTCGTGAGAACTGTATTGACACCCTTgtgt                 <  1:178347/292‑1 (MQ=255)`  
                                                                                                                                                                                                                                                                                         `aaagcaaTAGGAGAAATCAATAGGAGCGTTGCTGACATCGAAGCTAACGACCTCTT‑‑AGAGAGCGTCACTAAGTTCAACTGCGGGTTTATCTACGAATAATCAACCGCGGAGTACGTAAGCTACAGTCCGAGTGACTTCGGTGCGTATCTGGATGCGCTGGAAGCCGAGGTTGCACGAGGCGGTCTTATTGTGTTCCACAACGGTCACAAGTATGACGTTCCTGCATTGATCAAACTGGCAAAGTTGCAATTGAACCGAGAGTTCCACCTTCCTCGTGAGAACTGTATTGACACCCTTgtgt                 <  1:660541‑M2/294‑1 (MQ=255)`  
                                                                                                                                                                                                                                                                                          `atcaaacaGGAGAAATCAATATGATCGTTTCTGACATCGAAGCTAACGCCCTCTT‑‑AGAGAGCGTCACTAAGTTACACTGCGGGGTTATCTACGACTACTCCACCGCTGAGTACGTAAGCTACCGTCCGAGTGACTTCGGTGCGTATCTGGATGCGCTGGAAGCCGAGGTTGCACGAGGCGGTCTTATTGTGTTCCACAACGGTCACAAGTATGACGTTCCTGCATTGACCAAACTGGCAAAGTTGCAATTGAACCGAGAGTTCCACCTTCCTCGTGAGAACTGTATTGACACCCTTGtgtt                >  1:87280/8‑301 (MQ=255)`  
                                                                                                                                                                                                                                                                                            `tcaaTAGGAGAAATCAATATGATCGTTTCTGACATCGAAGCTAACGCCCTCTT‑‑AGAGAGCGTCACTAAGTTCCACTGCGGGGTTATCTACGACTACTCCACCGCTGAGTACGTAAGCTACCGTCCGAGTGACTTCGGTGCGTATCTGGATGCGCTGGAAGCCGAGGTTGCACGAGGCGGTCTTATTGTGTTCCACAACGGTCACAAGTATGACGTTCCTGCATTGACCAAACTGGCAAAGTTGCAATTGAACCGAGAGTTCCACCTTCCTCGTGAGAACTGTATTGACACCCTTGtgttgt              >  1:411227‑M2/5‑301 (MQ=255)`  
                                                                                                                                                                                                                                                                                            `gcaaTAGGAGAAATCAATATGATCGTTTCTGACATCGAAGCTAACGCCCTCTT‑‑AGAGAGCGTCACTAAGTTCCACTGCGGGGTTATCTACGACTACTCCACCGCTGAGTACGTAAGCTACCGTCCGAGTGACTTCGGTGCGTATCTGGATGCGCTGGAAGCCGAGGTTGCACGAGGCGGTCTTATTGTGTTCCACAACGGTCACAAGTATGACGTTCCTGCATTGACCAAACTGGCAAAGTTGCAATTGAACCGAGAGTTCCACCTTCCTCGTGAGAACTGTATTGACACCCTTGtgttgt              <  1:236042‑M2/297‑1 (MQ=255)`  
                                                                                                                                                                                                                                                                                             `caaTAGGAGAAATCAATATGATCGTTTCTGACATCGAAGCTAACGCCCTCTT‑‑AGAGAGCGTCACTAAGTTCCACTGCGGGGTTATCTACGACTACTCCACCGCTGAGTACGTAAGCTACCGTCCGAGTGACTTCGGTGCGTATCTGGATGCGCTGGAAGCCGAGGTTGCACGAGGCGGTCTTATTGTGTTCCACAACGGTCACACGTATGACGTTCCTGCATTGCCCAAACTGGCCAAGTTGCAATTGAACCGAGAGTTCCACCttcctcgtgagaactgtatctgtctcttatacacttt             >  1:49411‑M2/4‑283 (MQ=255)`  
                                                                                                                                                                                                                                                                                             `caaTAGGAGAAATCAATATGATCGTTTCTGACATCGAAGCTAACGCCCTCTT‑‑AGAGAGCGTCACTAAGTTCCACTGCGGGGTTATCTACGACTACTCCACCGCTGAGTACGTAAGCTACCGTCCGAGTGACTTCGGTGCGTATCTGGATGCGCTGGAAGCCGAGGTTGCACGAGGCGGTCTTATTGTGTTCCACAACGGTCACAAGTATGACGTTCCTGCATTGACCAAACTGGCAAAGTTGCAATTGAACCGAGAGTTCCACCTTCCTCGTGagaactgtattgacactgtctcttatac             >  1:489370‑M2/4‑289 (MQ=255)`  
                                                                                                                                                                                                                                                                                             `caaTAGGAGAAATCAATATGATCGTTTCTGACATCGAAGCTAACGCCCTCTT‑‑AGAGAGCGTCACTAAGTTCCACTGCGGGGTTATCTACGACTACTCCACCACTGAGTACGTAAGCTACCGTCCGAGTGACTTCGGTGCGTATCTGGATGCGCTGGAAGCCGAGGTTGCACGAGGCGGTCTTATTGTGTTCCACAACGGTCACAAGTATGACGTTCCTGCATTGACCAAACTGGCAAAGTTGCAATTGAACCGAGAGTTCCACCTTCCTCGTGAGAACTGTAttgacaccctgtctcttat             >  1:380530‑M2/4‑292 (MQ=255)`  
                                                                                                                                                                                                                                                                                                       `aaTCAATATGATCGTTTCTGACATCGAAGCTAACGCCCTCTT‑‑AGAGAGCGTCACTAAGTTCCACTGCGGGGTTATCTACGACTACTCCACCGCTGAGTACGTAAGCTACCGTCCGAGTGACTTCGGTGCGTATCTGGATGCGCTGGAAGCCGAGGTTGCACGAGGCGGTCTTATTGTGTTCCACAACGGTCACAAGTATGACGTTCCTGCATTGACCAAACTGGCAAAGTTGCAATTGAACCGAGAGTTTCACCTTCCTCGTGAGAACTGTATTGACACCCTTGTGTTGTCACGTTTGatt   <  1:448268/301‑1 (MQ=255)`  

                                                                                                                                                                                                                                                                                                         `|`                                                                                                                                                                                                                                                                                                               
`GGACAAGACAGACTGGCTGGAAGAAACCAGAAGTTACCAAGCTGAGTCATAAGGTGTTCGATAAGGACACTATGACCCACATCAAGGCTGGTGATTGGGTTAAGGTTGACATGGGAGTTGTTGGTGGATACGGCTACGTCCGCTCAGTTAGTGGCAAATATGCACAAGTGTCATACATCACAGGTGTTACTCCACGCGGTGCAATCGTTGCCGATAAGACCAACATGATTCACACAGGTTTCTTGACAGTTGTTTCATATGAAGAGATTGTTAAGTCACGATAATCAATAGGAGAAATCAATATGATCGTTTCTGACATCGAAGCTAACGCCCTCTT‑‑AGAGAGCGTCACTAAGTTCCACTGCGGGGTTATCTACGACTACTCCACCGCTGAGTACGTAAGCTACCGTCCGAGTGACTTCGGTGCGTATCTGGATGCGCTGGAAGCCGAGGTTGCACGAGGCGGTCTTATTGTGTTCCACAACGGTCACAAGTATGACGTTCCTGCATTGACCAAACTGGCAAAGTTGCAATTGAACCGAGAGTTCCACCTTCCTCGTGAGAACTGTATTGACACCCTTGTGTTGTCACGTTTGATTC  >  NC_001604.1/14051‑14647`

  

Alignment Legend

Aligned base mismatch/match (shaded by quality score): `ATCG/ATCG` `< 3 ≤` `ATCG/ATCG` `< 10 ≤` `ATCG/ATCG` `< 22 ≤` `ATCG/ATCG` `< 35 ≤` `ATCG/ATCG` `< 38 ≤` `ATCG/ATCG`

Unaligned base: `atcg`    Masked matching base: `atcg`    Alignment gap: `‑`    Deleted base: `‑`

Reads not counted as support for junction

read\_name Not counted due to insufficient overlap past the breakpoint.

read\_name Not counted due to not crossing MOB target site duplication.
